# Adherence to the Mediterranean Diet Among Primary School Children in the Tagus Lezíria Region, Portugal: A Study on Eating Habits

**DOI:** 10.3390/nu17172853

**Published:** 2025-09-02

**Authors:** Vanda Lopes de Andrade, Inês Ferrão, Maria Figueiredo, Igor Dias, Paula Pinto, Paula Ruivo

**Affiliations:** 1 School of Agriculture, Santarém Polytechnic University, Quinta do Galinheiro—S. Pedro, 2001-904 Santarém, Portugal; 200300105@esa.ipsantarem.pt (I.F.); 210300180@esa.ipsantarem.pt (M.F.); igor.dias@esa.ipsantarem.pt (I.D.); paula.pinto@esa.ipsantarem.pt (P.P.); 2 Research Centre for Natural Resources, Environment and Society (CERNAS), Santarém Polytechnic University, Quinta do Galinheiro—S. Pedro, 2001-904 Santarém, Portugal; 3 Life Quality Research Centre (CIEQV), Santarem Polytechnic University, Complexo Andaluz, Apartado 279, 2001-904 Santarém, Portugal; 4 Research Institute for Medicines (iMed.ULisboa), Faculty of Pharmacy, Universidade de Lisboa, 1649-003 Lisboa, Portugal

**Keywords:** Mediterranean diet, KIDMED index, Tagus Lezíria, eating habits, demographic factors, children, public health

## Abstract

**Background/Objectives:** Poor dietary habits remain a significant public health concern, highlighting the need to promote healthy and sustainable eating patterns, especially in childhood and adolescence. This study assessed the eating habits of primary school children in the Tagus Lezíria region, focusing on adherence to the Mediterranean Diet (MD), recognized for its health and environmental benefits. **Methods:** The KIDMED index (Mediterranean Diet Quality Index for children and adolescents) was applied to 435 children, the vast majority (97.7%) aged 6–10 years. **Results:** The data showed that 64.6% of participants exhibited high adherence to the MD, 32.0% medium, and 3.5% low adherence. Despite these encouraging levels, several gaps were identified: 80% of the children did not meet the recommendation for regular nut consumption (≥2–3 times/week), 44% consumed legumes less than once a week, and 43% failed to eat vegetables more than once a day. Regarding demographic factors, no significant differences were observed in overall adherence categories; however, differences emerged in specific eating behaviours. For example, girls consumed more vegetables and cereals than boys, and children in rural areas consumed less dairy than those living in urban settings. **Conclusions:** These findings emphasize the importance of reinforcing targeted school-based educational interventions to promote healthier dietary behaviours, particularly increased consumption of fruits, vegetables, fish, pulses, and nuts. Strengthening children’s adherence to the MD from an early age may contribute to improving their health and fostering sustainable eating practices.

## 1. Introduction

The current trend towards adopting Western dietary patterns in Europe is a growing concern, particularly among younger generations [1]. This shift is associated with increased consumption of processed and energy-dense foods, contributing to public health issues such as obesity and non-communicable diseases (NCDs). Obesity, a major public health concern, is itself a non-communicable disease and a leading risk factor for other NCDs, including Type 2 diabetes mellitus (T2DM) and cardiovascular diseases (CVDs) [2]. Collectively, NCDs—including CVDs, cancers, chronic respiratory diseases, and T2DM—are responsible for approximately 74% of global deaths [3]. Many of these diseases are driven by modifiable behavioural risk factors, with unhealthy diets being among the most significant [4,5]. A healthy diet includes adequate amounts and appropriate proportions of fruits, vegetables, legumes, nuts, and whole grains and limits the intake of free sugars, salt, saturated fats, and highly processed products. In children, sugary drinks are particularly associated with adverse health outcomes such as excessive weight gain and increased dental caries [6].

The Mediterranean Diet (MD) aligns closely with these healthy eating principles. According to Portugal’s National Program for the Promotion of Healthy Eating [7], the MD is characterized by the following: (1) frugality and simple cooking methods that preserve nutrients, such as soups and stews; (2) high intake of plant-based foods—vegetables, fruits, whole grains, legumes, nuts, and seeds; (3) preference for local and seasonal produce; (4) olive oil as the main fat source; (5) moderate consumption of dairy products; (6) use of herbs instead of salt for seasoning; (7) frequent consumption of fish and limited intake of red meat; (8) moderate wine consumption during meals; (9) water as the primary beverage; and (10) the cultural and social practice of sharing meals. Adopting the Mediterranean dietary pattern is associated with numerous health benefits, including reduced risk of CVDs, strokes, T2DM, and certain cancers [8,9]. The MD also meets the criteria of a sustainable diet, as defined by the Food and Agriculture Organization of the United Nations (FAO) in 2012 [10]: “Sustainable diets are protective and respectful of biodiversity and ecosystems, culturally acceptable, accessible, economically fair, and affordable; nutritionally adequate, safe, and healthy; while optimizing natural and human resources” [11,12]. It is evident that sustainable diets, such as the MD, contribute to a healthier environment, which in turn positively impacts public health [13,14,15]. However, there is a growing trend towards abandoning this traditional dietary pattern in Mediterranean countries, even among the populations historically associated with it [16,17,18]. In Portugal, longitudinal studies have shown an increase in adherence to the MD among adults during the pandemic confinement periods (2019–2021); this, however, was not fully sustained after the end of the pandemic [19]. Moreover, studies conducted in several countries showed a significant decline in adherence to the MD among children and adolescents, with a notable increase in the consumption of processed foods and a decrease in the intake of fruits, vegetables, and fish [20,21,22]. The reasons for this trend of low adherence to the Mediterranean diet have been recently discussed, with a focus on socioeconomically disadvantaged groups and other demographic factors [17,23,24,25,26]. Nevertheless, there is a worrying lack of information about the nutritional status of primary school-aged children, even though nutrition during school years is critical to support healthy growth trends until maturity [27]. Inadequate nutrition negatively impacts the physical and neurocognitive development of children aged 5 to 10 years, with long-term repercussions. The consumption of fast food and sugary beverages has been associated with behavioural problems, poor concentration, obesity, and emotional development issues [27]. Conversely, healthy diets have been shown to improve children’s cognitive abilities, such as concentration and memory, as well as mood, energy levels, and academic performance [28]. In Portugal, few studies have addressed adherence to the MD or the healthy food choices of primary school-age children. At the national level, a survey undertaken in 2016–2017, with 521 children (3 to 9 years), and 632 adolescents (10–17 years) reported a low level of adherence to the MD in these groups [29]. A more recent study performed with 295 children 7 to 11 years old, from 20 schools within a northern Portugal city (Porto) similarly reported low adherence to the MD, mainly due to an insufficient consumption of nuts, whole grains, legumes, and fish [30]. To our knowledge, no data exist from other specific regions of Portugal regarding MD adherence of primary school-aged children.

Thus, this study aims to contribute to increasing the knowledge on MD adherence, specifically by assessing adherence to the Mediterranean Diet among primary school children from the Tagus Lezíria region, in Portugal, and to explore the influence of demographic factors on this adherence. Understanding these determinants may support the development of public policies and educational programs that are better tailored to local contexts, filling a gap of information about this age group in this region.

## 2. Methods

### 2.1. Ethics and Sampling

This study was approved by the IPSantarém Ethics Committee in accordance with the guidelines of the Declaration of Helsinki (approval nº 37/2023; 20 December 2023). This study was conducted in collaboration with the Intermunicipal Community of Lezíria do Tejo (CIMLT), which proposed 19 primary schools across 11 municipalities in the Lezíria do Tejo region for interviews with pupils. Each School Board selected classes from the 1st to the 4th grade to take part in this study, and the teachers distributed informed consent letters to the pupils’ parents. Only children whose parents delivered a signed consent letter were eligible for this study. Of the 872 parents contacted, 435 returned signed informed consent forms authorizing their child’s interview. The interviews were conducted in-person by trained professionals, during the months of January and February in 2024.

### 2.2. Adherence to Mediterranean Diet

Adherence to the MD was assessed through the interviews, using the KIDMED questionnaire, originally developed for children and adolescents in Spain [31] and later validated in a Portuguese sample [32]. The KIDMED questionnaire consists of 16 yes-or-no questions, where positive responses score +1 or −1 depending on their alignment with MD principles, resulting in a final score between 0 and 12. Higher scores indicate stronger adherence. Scores can be categorized into three levels: (1) High Adherence: ≥8; (2) Medium Adherence: 4–7; and Low Adherence: ≤3 [32]. To explore the influence of demographic factors on the adherence of children to the MD, the interviews included inquiries about the participants’ sex, age, and area of residence (whether rural or urban).

### 2.3. Statistical Analysis

Data were analysed using SPSS version 26. Absolute and relative frequencies were calculated to characterize the sample, assess KIDMED Index scores, and describe responses to each questionnaire item. Non-parametric hypothesis tests were applied to compare groups regarding the influence of demographic parameters on adherence to the MD, using Mann–Whitney and Kruskal–Wallis tests for scale and ordinal variables, while Chi-square tests were used for nominal variables.

## 3. Results

### 3.1. Demographic Characteristics of the Sample

The study sample included a total of 435 participants, with 45.7% boys and 54.3% girls. Participants were aged between 6 and 13 years, with a mean age 8.19 ± 1.10 years. The majority of children were 7-, 8-, and 9-years old, representing 84.2% of the participants (Table 1). The analysis of the areas of residence revealed a higher concentration of participants in rural areas, representing 58.2% of the sample, while the remaining children, 41.6%, lived in urban areas (Table 1).

### 3.2. Adherence to the Mediterranean Diet

**Figure 1:**
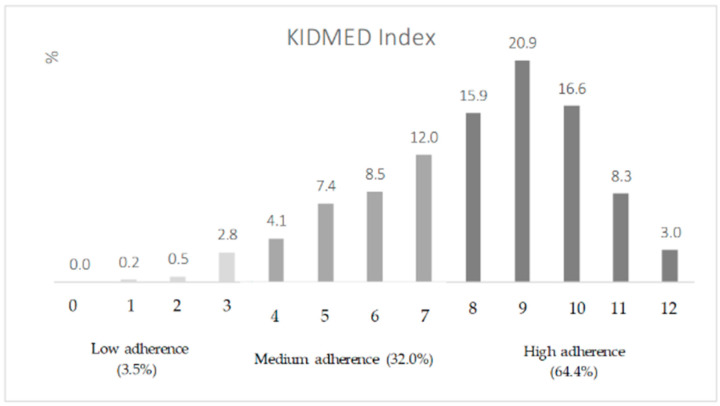
Adherence to the Mediterranean Diet evaluated by the KIDMED Index (Mediterranean Diet Quality Index to evaluate MD adherence among children and adolescents. The KIDMED Index ranges from 0 to 12, with scores of 0–3 indicating low adherence, 4–7 medium adherence, and 8–12 high adherence. The bars represent the percentage of the inquired children in each index score.

Figure 1 shows the percentage distribution of adherence to the MD evaluated by the KIDMED Index across the sample. The average KIDMED Index was 8.0 ± 2.2 with one student scoring the minimum of one point and thirteen students achieving the maximum of twelve points. The scores are also divided into three main adherence categories: low (score ≤ 3), medium (scores 4 to 7), and high (scores ≥ 8). It can be noted that 64.60% of the respondents were classified as having “high adherence,” 32.0% as “medium adherence,” and 3.5% as “low adherence” (Figure 1). In the “high adherence” category, the most frequent score was 9, representing approximately 1/5 of all participating children, 20.9%, and about one-third of those which fitted this category (32.38%) (Figure 1). The “medium adherence” category is concentrated at the upper limit of the range (score 7), being represented by 12.0% of the total respondents and 37.4% of those which matched in this category.

When analysing each question of the KIDMED Index, the results show that 80% or more of the children met the MD principles for 10 of the 16 questions of the KIDMED questionnaire (Table 2). The highest alignment with MD principles occurs with the use of olive oil at home, with 92.4% of the children answering “Yes”. Lower percentages were found regarding queries concerning “Second serving of fruit daily”, “Fresh or cooked vegetables > 1/day”, “Regular fish consumption (at least 2–3/week)”, “Pulses > 1/week”, and “Two milk glass/yoghurts and/or 40 g cheese daily”, for which the percentages of answers in alignment with the MD varied between 56.8 and 69.8%. Of notice is the lowest percentage found for the question “Regular nut consumption (at least 2–3/week)”, which showed only 20.5% of children meeting the recommendation (Table 2).

### 3.3. Influence of Demographic Factors on Adherence to the Mediterranean Diet

Regarding the three MD adherence categories (low, medium and high), no significant differences were observed between boys and girls (*p* = 0.177), among ages from 6 to 10 (*p* = 0.860), or between urban and rural residence (*p*= 0.270). When analysing the influence of the demographic factors, sex, age, and residence, per KIDMED question, some significant differences were found.

**Figure 2:**
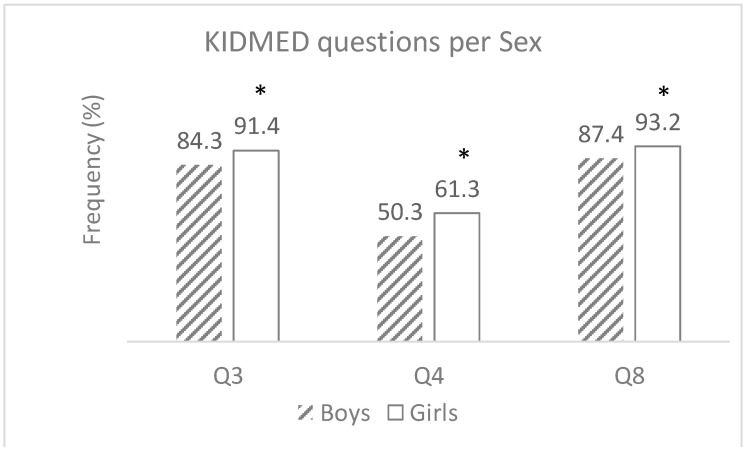
KIDMED index questions for which a significant difference was found between boys and girls: Q3: consumption of fresh or cooked vegetables daily; Q4: consumption of fresh or cooked vegetables > 1/day; and Q8: consumption of pasta or rice almost daily (≥5 days/week). The bars represent the percentage of the inquired children that meet the MD principles (responding “Yes” to the questions). Significant differences were assessed by Chi-square tests; * indicates significant difference (*p* < 0.05).

Significant differences between girls and boys were observed in the consumption of two types of foods: vegetables, and cereals at the main meals, with girls showing a higher consumption than boys. In fact, the percentages of girls consuming “Fresh or cooked vegetables daily” (question 3, Q3) and “Fresh or cooked vegetables > 1/day” (question 4, Q4), were 91.4% and 61.3%, respectively, values significantly higher than the percentages found for boys, which were 84.3% (*p* < 0.001) and 50.3% (*p* = 0.021), respectively (Figure 2). A similar picture was observed regarding the consumption of “Pasta or rice almost daily (≥5 days/week)” (question 8, Q8), with the percentage of girls represented at 93.2%, compared to 87.4% of boys (*p* = 0.011) (Figure 2).

**Figure 3:**
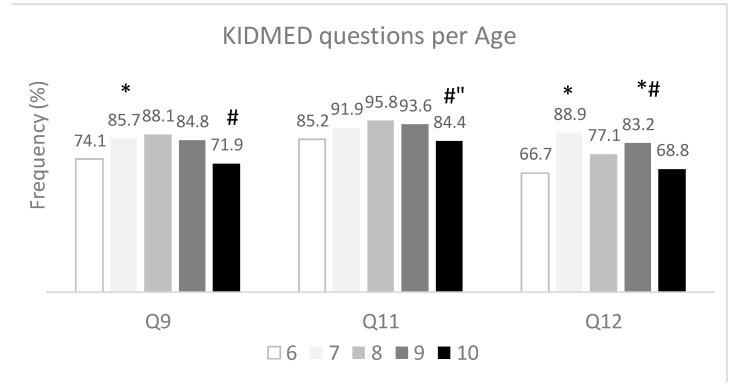
KIDMED questions showing significant differences by ages. Q9, Q11, and Q12 represent the questions pertaining to the consumption of “Cereal or cereal product for breakfast”, “Use of olive oil at home/day”, and “No breakfast”, respectively. Bars represent the percentage of children meeting the Mediterranean Diet principles (children responding “Yes” to Q9 and Q11 and “No” to Q12). Differences were assessed by Chi-square tests; *, # and ″ indicate significant difference (*p* < 0.05) from the 6-, 8-, and 9-year-old children, respectively.

Regarding the influence of children’s age on the frequency of consumption of the foods included in the KIDMED questionnaire, significant differences were found in the questions related to breakfast and olive oil use. For the question “Do you eat cereals or cereal-based products for breakfast?” (question 9, Q9), significant differences were observed between the children of 6 and 7 years old and between the children of 8 and 10 years old (Figure 3). A higher percentage of the 7-year-old children answered “yes” (85.7%), compared to the 6-year-old children (74.1%; *p* = 0.048), and a higher percentage of the 8-year-old children answered yes (88.1%) compared to the 10-year-old children (71.9%, *p* = 0.013 (Figure 3). Still, concerning breakfast, the habit of not skipping breakfast (question 12, Q12) showed the highest percentages in the 7- and 9-year-old children (88.9% and 83.2%, respectively). The lowest percentage was observed for the 6-year-old children (66.7%), which differed significantly from the 7-year-old (*p* = 0.009) and the 9-year-old children (*p* = 0.010) (Figure 3). The use of olive oil (question 11, Q11) had the lowest % in the group of children, with 10 year olds (84.4%) exhibiting a significant difference compared to the 8- or 9-years-old groups (*p* = 0.022 and *p* = 0.041), represented by 95.8 and 93.9%, respectively (Figure 3).

**Figure 4:**
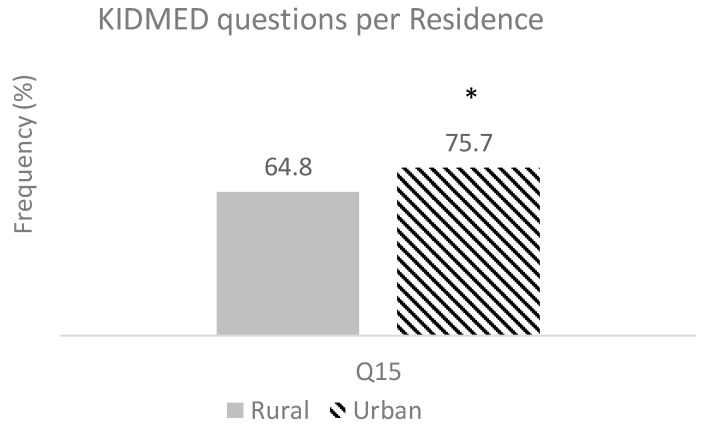
KIDMED question showing significant difference between rural and urban residences. (question 15, Q15) (representing the consumption of “Two milk glass/yoghurts and/or 40 g cheese daily”.) Bars represent the percentage of children meeting the MD principles (responding “Yes” to Q15). Differences were assessed by Chi-square tests; * indicates significant difference (*p* < 0.05) from the rural children.

The other demographic factor studied was the influence of living in rural or urban settings on MD adherence. A significant difference (*p* = 0.036) was found regarding the consumption of dairy products, with a lower percentage of children living in rural areas reporting daily consumption these products. In fact, for the question “Do you drink 2 glasses of milk/yogurt or eat 1 large slice of cheese daily?” (question 15, Q15), 64.8% of children in rural areas reported consuming these amounts daily, compared to 75.7% in urban areas (Figure 4).

## 4. Discussion

A major concern during childhood and adolescence is the early abandonment of a Mediterranean dietary pattern, while the Western diet (rich in saturated fats, refined carbohydrates, and salt) is gaining popularity, contributing to the rising incidence of NCDs, particularly obesity [33,34]. Children spend most of their day at school and most of them eat lunch, one of their main meals, at school. It is estimated that approximately 40% of their total energy intake is consumed in schools [34]. As dietary habits are formed during childhood and tend to be consolidated in adulthood [34], understanding the eating habits of children is essential to gain a general perspective and to identify areas that need improvement, particularly through subsequent educational interventions. Under such a perspective, we evaluated general adherence to the MD among 435 primary school children from a group of schools in Tagus Lezíria, in Portugal, analysed their food habits according to the recommendations of the MD, and explored the influence of demographic factors on MD adherence and food habits.

### 4.1. Adherence to the Mediterranean Diet

Interestingly, our study showed an average KIDMED index of 8.0 for primary school-aged children, which is classified as a high level of adherence (KIDMED index ≥ 8). Moreover, 64.6% of the children participating in our study were classified as having a “high adherence” (Figure 1). This contrasts with what has been reported in other Mediterranean countries. A systematic review of cross-sectional surveys conducted in southern European countries in 2016 (Greece, Cyprus, Spain, and Italy) reported that roughly half of the children and adolescents participating in the survey had moderate adherence to the MD (measured by the KIDMED or any other score), while nearly half may have low adherence, with a trend toward poorer adherence over time [35]. Spain showed a higher percentage of children and adolescents classified as high adherence (46.4%), while the other countries had low percentages of high adherence (ranging from 4 to 10%). A more recent study in Greece (2018–2022 national survey with children 6 to 9 years old) reported 22.7% of children classified as high adherence by the KIDMED index, 37.6% as medium adherence, and 39.7% as low adherence [36].

It seems that studies developed in Portugal present a trend for higher MD adherence levels of children, when compared with the mentioned European Mediterranean countries. In fact, a study undertaken in public elementary schools in the city of Guimarães (North of Portugal) in 2007–2008 showed that most children (69.1%) reported medium adherence to the MD [26], and a study performed in 2018, in private elementary schools in the municipalities of Maia and Porto (also in the North of Portugal), reported a high level of adherence (≥8 points) in 77.6% of the sample [37]. Taking the fact that in the study performed 17 years ago, less than 5% of the children scored as high adherence to the MD [26], and that in the study of 2018 [37] and our study, conducted in 2024, the percentages of high adherents were above 50%, it may be suggested that MD adherence in children has been increasing in Portuguese primary school children. This is in line with the results obtained with a national survey distributed to the Portuguese adult population, which showed an increase of 15% in adherence to the MD from 2016 to 2020 [19].

Regarding the different items assessed in the KIDMED questionnaire, the study performed in Maia and Porto, Portugal, by Marques et al. (2019) [37], underlines the habit of having breakfast every day, the use of olive oil, and the consumption of at least one piece of fruit per day, as well as raw or cooked vegetables, dairy products, fish, and cereals and their products. These results are in alignment with the results of our study, except for fish consumption, as lower percentages were found in our study regarding “Regular fish consumption (at least 2–3/week)”. It is a possibility that this difference can be explained by the higher proximity of Maia and Porto to the sea, when compared to the sampled areas of Tagus Lezíria. A common issue is the high use of olive oil, and indeed, Portugal’s olive oil production is now five times higher than it was in the early 2000s [38], making them more available. Of notice is the fact that in the same way as Marques et al. (2019) [37], a “Second serving of fruit daily” exhibited a low frequency. We also highlight the lowest percentage found for the question pertaining to “Regular nut consumption (at least 2–3/week)”, which was 20.5% (Table 2). This seems to be one of the foods included in the MD that have the lowest adherence in general, as it was reported also for Portuguese adults in 2020 (61% of the sample did not consume the recommended portion of nuts) [39].

### 4.2. Influence of Demographic Factors on Adherence to the Mediterranean Diet

Marques et al. (2019) [37], after analysing the responses by gender in the study performed in Maia and Porto, found that they were equivalent between boys and girls. In our study, girls exhibited a trend for a slightly more favourable distribution regarding the KIDMED Index, when compared to boys, but with no statistical significance. Such a lack of marked differences between sexes might be attributable to the low age of the studied population. It is a possibility that this slight difference can reflect a trend that might appear with adolescence, since a study with children aged 10 to 13 years from both Spain and Germany showed that females reached higher results in KIDMED nutritional scores [40]. This may be related to greater body image concerns among girls, as evidence suggests that although both boys and girls experience body dissatisfaction, girls are more likely to be dissatisfied with their appearance and weight [41,42]. Despite a lack of significance in the KIDMED index between sexes, our study found significant differences in some individual questions of the KIDMED index. Specifically, the proportion of girls consuming fresh or cooked vegetables daily, and more than once per day, and consuming pasta or rice almost daily was significantly higher than boys (*p* < 0.001; *p* = 0.021 and *p* = 0.011, respectively). The results of the study by Marques et al., 2019 [37] were somewhat different, with girls consuming significantly more fruit, fish, and legumes than boys. Taken together, these findings suggest that the eating patterns of girls and boys at school age are different, but these differences do not necessarily translate into distinct overall MD adherence levels.

With regard to age, our study did not reveal significant differences in MD adherence. Nevertheless, it was observed that children aged 10 showed the lowest adherence to the MD. The trend was observed in a study in Greece, where increased age was negatively associated with KIDMED scores [35]. However, a study performed to evaluate the differences in the Mediterranean diet and its components among primary and secondary school children and adolescents living in northern Italy reported that some unhealthy behaviours were more prevalent in younger children [21]. When looking at the responses of the children to the individual questions of the KIDMED questionnaire, we observed significant differences in the questions regarding breakfast habits, with both younger (6 year olds) and older (10 year olds) children having the lowest percentages of participants meeting the requirements of a healthy breakfast.

Concerning the residence zone, although statistical significance was not reached, the rural group was less represented in the “high adherence” category than the urban children (60.5 and 70.2%, respectively, Figure 4). Furthermore, the percentage of children living in rural areas that consumed the recommended portions of dairy products daily was significantly lower than those that lived in urban settings (*p* = 0.036). Similar findings were reported in Greece and Italy [35]. Such disparities may be partly explained by multiple barriers that create challenges for rural residents: reduced access and use of reliable health information, geographic isolation, distance, limited financial resources, and fewer health care services [43]. However, contrary results were reported in Spain [35], where higher MD adherence in children living in rural areas was reported. The contradictory results from different studies and different countries highlight the complexity of assessing the influence of demographic factors such as sex, age, and residence area in the adherence of school-age children to the MD. In contrast, more consistent results were obtained for other factors such as economic status, physical activity levels, and maternal education, which showed positive associations with MD adherence in children [35,41,44].

### 4.3. Practical Implications

Although no complex or detailed interpretations can be drawn regarding the influence of demographic factors on the consumption of specific food items, these findings are undoubtedly of crucial relevance at a regional scale, in this case the Tagus Lezíria region, as they can help address particular situations by enabling parents and educators to promote healthier habits and encourage the consumption of specific food items tailored to the needs of specific groups.

Several national programs to promote healthier food choices at schools are ongoing, such as the following: free distribution of fruit, vegetables, and milk in schools; the implementation of guidelines to regulate school meals, bars, and vending machines; and the restriction of high-sugar and high-salt products while reinforcing access to healthier options [45]. Despite these efforts, our study emphasizes the need for interventions to change children’s behaviours towards the MD, specifically by promoting the importance of eating fruit and vegetables more than once a day, increasing fish consumption, and, above all, the consumption of nuts, which remains the least consumed food group within the MD recommendations. A recent meta-analysis of healthy lifestyle-based interventions targeted to children have shown improvements in MD adherence after these interventions, particularly when both children and parents were involved [46]. Therefore, implementing and consolidating such interventions across Portuguese public schools may represent a valuable strategy to foster healthier dietary habits from an early age.

### 4.4. Limitations of This Work

Despite all the cautions of the researchers to minimize bias, during questionnaire administration, several challenges inevitably arise when children are asked to report their eating habits:

Limited awareness of quantities and frequencies by the children (questions 2, 4, 5, 7, 10, and 15): Questions involving concepts of quantity or frequency, such as “two or more pieces of fruit,” “regularly,” or “at least 2 to 3 times a week” were sometimes difficult for children to interpret. This difficulty is closely related to how young children perceive time. Studies show that children estimate time accurately only when they are forced to focus on it, either through the experience of their actions’ duration or the frustration of unmet needs [47]. In the absence of such stimuli, time tends to hold little relevance in their daily routines. Moreover, before the age of 10, most children do not spontaneously use explicit time-control strategies, such as daily planning or monitoring the time spent on tasks [48], which further complicates their ability to report dietary frequencies.

Lack of knowledge about certain foods and classifications (questions 7, 10, and 15): Terms like “legumes,” “nuts,” and “dairy products” were not familiar to some young children, leading to confusion in interpreting the questions.

Disconnect from actual eating behaviour (questions 1, 3, 8, and 11): Questions about foods that may be part of a routine but go unnoticed, such as “Do you use olive oil at home?” or “Do you eat fish regularly?” may not elicit precise answers. Children might not be aware that olive oil is used in cooking, and they might eat foods like fish sticks without realizing they contain fish. This is often because processed foods, presented differently from their original ingredients, are not recognized by children as belonging to a specific food group.

Difficulty reporting less healthy behaviours (questions 6, 13, 14, and 16): Questions about consuming fast food, pastries, and sweets can be particularly challenging for children, especially if these foods are perceived negatively. Children may hesitate to respond honestly about behaviours they know are less healthy, leading to socially desirable answers.

Limited food autonomy (all questions): Most children aged 6 to 10 lack full autonomy over their food choices. Meals are typically prepared by parents or schools, which limits the child’s knowledge of what they are eating and how often. As a result, their answers may not accurately reflect their actual eating behaviour.

## 5. Conclusions

This study of adherence to the MD among children from primary schools in the region of Tagus Lezíria, in Portugal, showed that 64.6% of the participants presented a high level of adherence, based on the KIDMED index. This is a positive finding and consistent with the results from other Portuguese studies, suggesting that many children in this region maintain key aspects of a healthy dietary pattern. However, demographic factors such as gender, age, and area of residence revealed certain aspects that deserve attention. The data suggest that girls tend to have slightly better dietary habits than boys in some specific aspects of the KIDMED questionnaire, but no significant differences were found in overall adherence. Similarly, children from urban areas showed higher adherence than those in rural areas, although this difference did not reach statistical significance. Of greater concern is that considerable proportions of children did not meet the MD recommendations for key food groups, namely fruit (two or more servings per day), vegetables, fish, pulses, and especially nuts, which was the least consumed group. These results suggest the need for tailored interventions to change food behaviours in school-age children. It is important to emphasize that these results should be interpreted with caution due to specific limitations related to the age of the respondents, including difficulties in reporting portion sizes, frequency of consumption, and awareness of food items used to prepare meals. The results highlight the importance of continuing strategies to promote the MD, and it is crucial to implement educational programs tailored to the current reality, to promote a return to the key principles of the MD, and to ensure that high adherence to this healthy dietary pattern is maintained and strengthened.

## Figures and Tables

**Table 1:** Participants’ demographic characteristics. Data are expressed as frequencies (%) and sample size (N). Age is expressed as mean± standard deviation (mean ± sd) and N.

Sex (%/N)	
Boys	45.7/199
Girls	54.3/236
Age (mean ± sd/N)	8.2 ± 1.1/433 *
Age categories (%/N)	
6 years	6.4/28
7 years	19.8/86
8 years	32.2/140
9 years	32.2/140
10 years	7.8/34
11 years	0.7/3
12 years	0.2/1
13 years	0.2/1
Residence (%/N **)	
Rural	58.2/253
Urban (city/town/village)	41.6/181

* N for age = 433 due to missing data for 2 participants. ** N for rural area = 253 due to missing data for 1 participant.

**Table 2:** Participants’ answers, according to their alignment with Mediterranean Diet principles, to each question of the KIDMED questionnaire. Data are expressed as percentages (%) and number of children (N).

KIDMED Question	Answer	%/N
1. Fruit or fruit juice daily	(yes)	88.6/325
2. Second serving of fruit daily	(yes)	61.6/229
3. Fresh or cooked vegetables daily	(yes)	89.5/332
4. Fresh or cooked vegetables >1/day	(yes)	58.2/216
5. Regular fish consumption (at least 2–3/week)	(yes)	67.1/249
6. >1/week fast-food (hamburger) restaurant	(no)	85.4/316
7. Pulses >1/week	(yes)	56.8/210
8. Pasta or rice almost daily (≥5/week)	(yes)	90.0/334
9. Cereal or cereal product for breakfast	(yes)	84.1/312
10. Regular nut consumption (at least 2–3/week)	(yes)	20.5/76
11. Use of olive oil at home	(yes)	92.4/342
12. No breakfast	(no)	79.8/296
13. Dairy product for breakfast	(yes)	87.3/324
14. Commercially baked goods or pastries for breakfast	(no)	85.7/318
15. Two milk glass/yoghurts and/or 40 g cheese daily	(yes)	69.8/259
16. Sweets and candy several times a day	(no)	88.1/327

KIDMED Index (Mediterranean Diet Quality Index to evaluate MD adherence among children and adolescents). Criteria to meet MD principles are shown for each question as “yes” or “no”.

## Data Availability

The original contributions presented in this study are included in the article. Further inquiries can be directed to the corresponding authors.

## Abbreviations

The following abbreviations are used in this manuscript:

MD	Mediterranean Diet
KIDMED index	Mediterranean Diet Quality Index for children and adolescents
NCDs	Non-communicable diseases
T2DM	Type 2 diabetes mellitus
CVDs	Cardiovascular diseases
